# Unveiling molecular mechanisms of strobilurin resistance in the cacao pathogen *Moniliophthora perniciosa*

**DOI:** 10.1016/j.isci.2025.113180

**Published:** 2025-07-23

**Authors:** Paula F.V. Prado, Caio V.C. Mendes, Bárbara A. Pires, Gabriel L. Fiorin, Piotr Mieczkowski, Gonçalo A.G. Pereira, Paulo J.P.L. Teixeira, Daniela P.T. Thomazella

**Affiliations:** 1 Institute of Biology, University of Campinas (UNICAMP), Campinas, São Paulo 13083970, Brazil; 2 Department of Biology, “Luiz de Queiroz” College of Agriculture (ESALQ), University of São Paulo (USP), Piracicaba, São Paulo 13418900, Brazil; 3 Department of Genetics, University of North Carolina at Chapel Hill (UNC), Chapel Hill, NC 27514, USA; 4 Department of Genetics, “Luiz de Queiroz” College of Agriculture (ESALQ), University of São Paulo (USP), Piracicaba, São Paulo 13418900, Brazil

**Keywords:** Mycology, Plant biology, Interaction of plants with organisms, Plant pathology

## Abstract

Strobilurin fungicides inhibit mitochondrial respiration, leading to ATP depletion and oxidative stress. Although widely used in agriculture, these chemicals are ineffective against the cacao pathogen *Moniliophthora perniciosa*. Here, we show that *M. perniciosa* tolerates high concentrations of the commercial strobilurin azoxystrobin *in vitro*. Transcriptomic analysis revealed that short-term exposure triggers upregulation of genes related to catabolic pathways, including the glyoxylate cycle and fatty acid degradation, alongside repression of genes involved in anabolic processes, such as cell division and ribosome biogenesis. Simultaneously, genes associated with cellular detoxification and oxidative stress responses were strongly induced. These alterations suggest that *M. perniciosa* remodels its metabolism to counteract fungicide toxicity. Remarkably, long-term exposure to azoxystrobin led to the emergence of a resistant mutant harboring mutations in two putative growth and transcriptional regulators. This work provides new insights into the molecular basis of strobilurin resistance and informs strategies for more effective fungicide deployment in agriculture.

## Highlights

• *Moniliophthora perniciosa* tolerates high concentrations of azoxystrobin
• Azoxystrobin induces transcriptional changes indicative of metabolic reprogramming
• Prolonged azoxystrobin exposure led to the emergence of a resistant mutant
• Mutations in growth and transcriptional regulators may explain the resistance trait

## Graphical abstract

**Figure:**
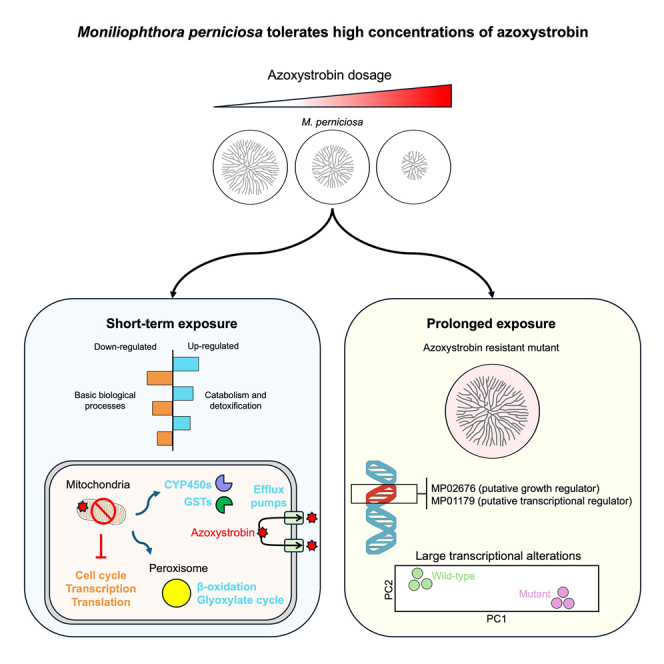

## Introduction

The establishment and progress of human civilizations have been closely tied to the development of agriculture. Not surprisingly, plant diseases have been a main concern for humankind since ancient times.^1^ Major disease outbreaks, such as late blight, responsible for the Irish Potato Famine in the 19th century, or the Panama disease, which threatened the global banana production in the 1950s, have shown how plant pathogens can greatly impact our society, changing the course of human history.^2^^,^^3^ Currently, many other agronomically important crops are affected by pathogens, which cause significant yield losses and constraints to the global food supply.^4^ Thus, disease control is essential for ensuring efficient agricultural production.

We have battled the impact of pathogens in agriculture mostly through the cultivation of resistant plant varieties and/or the use of agrochemicals. However, continuous genetic changes within pathogen populations have often led to the loss of previously effective strategies for disease control. Resistant cultivars have historically been developed through selection and breeding programs. More recently, genetic manipulation based on biotechnological tools (i.e., genome editing technologies) has emerged as a promising alternative to classical breeding, which can be a time-consuming process. Yet, the use of genetic engineering to develop plants with improved agronomic traits is still subject to regulatory issues and public acceptance in many countries.^5^^,^^6^ In addition to resistant varieties, agrochemicals have ensured an adequate food supply for many years and are particularly important for crops that are difficult to breed (e.g., banana and cassava). However, the indiscriminate use of these molecules can be highly harmful to both the environment and human health.^7^ Additionally, continued use of chemicals often selects for resistant/tolerant phytopathogens.^8^

The emergence of fungicide resistance in pathogen populations due to their extensive and long-term use is well-illustrated by strobilurin fungicides. Isolated from the basidiomycete *Strobilurus tenacellus*, strobilurin was modified for commercial use and promptly became one of the major classes of agricultural fungicides worldwide.^9^ The significant impact of these molecules on agriculture is exemplified by the synthetic strobilurin azoxystrobin, which is one of the most used fungicides in agriculture.^10^ Strobilurins belong to the quinol oxidation inhibitor (QoI) family, a class of molecules that inhibit mitochondrial respiration by specifically binding to the Qo domain of mitochondrial cytochrome *bc*_1_ (complex III).^9^ These chemicals effectively reduced agricultural losses for several decades. However, one of their most prominent advantages, the highly precise mode of action, also proved to be their major limitation.^9^ Single-point mutations in the gene encoding cytochrome *b* (*Cytb*), which is part of the strobilurin-binding site at complex III, led to the emergence of fungal strains resistant to these highly specific inhibitors.^11^

Despite their effectiveness in controlling multiple plant pathogens, strobilurins have been largely ineffective against the witches’ broom disease (WBD) of cacao. WBD is one of the major phytopathological problems that impact the production of cocoa beans in the Americas and has caused devastating socioeconomic consequences, especially in Brazil.^12^ This disease is caused by the basidiomycete *Moniliophthora perniciosa,* and little is known about the molecular mechanisms underlying strobilurin tolerance in this fungus. The first insights on *M. perniciosa* tolerance to strobilurin were provided by Thomazella et al. (2012),^13^ who identified a single copy of an alternative oxidase gene (*Mp-Aox*) in the fungal genome.^14^ Alternative oxidase (AOX) is the main enzyme of the alternative respiratory pathway and can limit the effectiveness of strobilurins *in planta*.^11^ Consistently, Thomazella et al. (2012) demonstrated that Mp-AOX plays an important role in strobilurin tolerance.^13^

Beyond AOX, additional mechanisms have been proposed in other fungal species. For example, several fungi, including *Plasmopara viticola*, *Colletotrichum gloeosporioides*, *Botrytis cinerea*, and *Cercospora nicotianae*, can mitigate strobilurin toxicity through the upregulation of efflux pumps.^15^ Furthermore, reactive oxygen species (ROS) scavenging has been suggested to enhance strobilurin tolerance in *Corynespora cassiicola*.^16^ Despite these findings, the broader molecular landscape of QoI fungicide resistance in fungi remains poorly understood. Therefore, understanding the mechanisms associated with fungal response to these chemicals may lead to the identification of new molecular targets associated with strobilurin tolerance/resistance. Co-inhibition of such targets could completely halt fungal development and prevent the emergence of strobilurin-resistant strains, ultimately increasing fungicide efficiency and durability.

Here, we demonstrate that *M. perniciosa* tolerates unusually high concentrations of strobilurins *in vitro*, and this tolerance is not due to the typical mutations in the fungal *Cytb* gene. A time-course analysis of the fungal transcriptome following fungicide exposure revealed that, in addition to the upregulation of *Mp-AOX*,^13^ the pathogen undergoes intense metabolic reprogramming to counteract the effects of respiratory chain inhibition, including reduced ATP production and increased oxidative stress. These alterations involve the upregulation of genes associated with the mitochondrial respiratory chain, tricarboxylic acid cycle, glyoxylate cycle, and fatty acid and amino acid catabolism, which may compensate for the reduced ATP production. Additionally, increased expression of genes encoding efflux pumps and detoxifying enzymes may contribute to fungal tolerance to strobilurin by alleviating oxidative stress. Finally, we show that long-term exposure of *M. perniciosa* mycelia to azoxystrobin led to the emergence of a resistant mutant with a markedly distinct transcriptional signature when compared to wildtype isolates. Mutations introducing premature stop codons in two genes encoding putative growth and transcriptional regulators may have driven the extensive transcriptional alterations observed in the mutant, contributing to its increased strobilurin resistance. Taken together, our results provide valuable insights for the rational use and development of durable fungicide-based strategies to control WBD and possibly other major fungal diseases worldwide.

## Results

### *M. perniciosa* tolerates high concentrations of strobilurins

**Figure 1:**
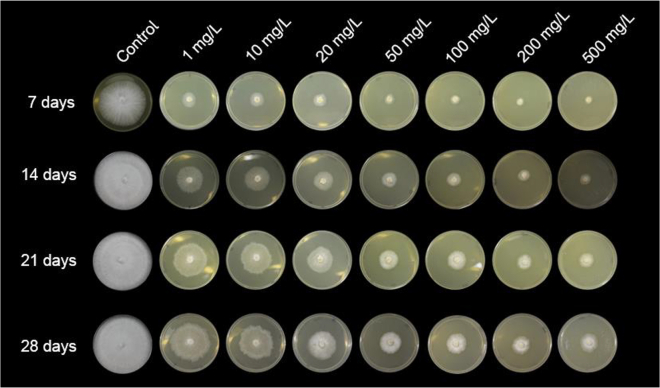
Evaluation of *M. perniciosa* growth in the presence of the strobilurin azoxystrobin Cultivation of *M. perniciosa* under different concentrations of azoxystrobin for 28 days. Growth was observed at all tested concentrations. Although the fungal growth rate was affected in a dose-dependent manner, even concentrations as high as 500 mg/L were not sufficient to completely halt *M. perniciosa* growth. Representative images from three replicates are shown. See also Figure S2.

Azoxystrobin is a synthetic strobilurin, with a recommended field dosage ranging from 40 mg/L to 120 mg/L of active ingredient (Amistar WG, Syngenta). We evaluated *M. perniciosa* growth in culture media containing increasing concentrations of this fungicide (ranging from 1 mg/L to 500 mg/L) over a 28-day period. While a clear reduction in the *M. perniciosa* growth rate was observed, none of the concentrations tested fully inhibited fungal development (Figures 1 and S2A). As expected, *M. perniciosa* growth was more strongly suppressed at higher concentrations. However, the fungus was still able to grow even at concentrations as high as 500 mg/L (Figures 1 and S2A). This ability to tolerate high azoxystrobin concentrations was also observed in additional *M. perniciosa* isolates from different biotypes (Figure S2B), suggesting a conserved tolerance mechanism.

Interestingly, even after prolonged exposure (60 days) to the fungicide in solid media, *M. perniciosa* was able to recover its normal growth rate when transferred to media without fungicide, demonstrating that azoxystrobin treatment does not cause complete mycelial death. Furthermore, *M. perniciosa* also exhibited high tolerance to other two synthetic strobilurins (metominostrobin and picoxystrobin, Figure S2A). Remarkably, the *M. perniciosa* isolate used in this experiment (FA553) does not contain any of the typical mutations in the *CytB* gene that are known to confer resistance to strobilurins in other fungi (Figure S3).^11^^,^^17^^,^^18^ Therefore, other mechanisms may contribute to the high tolerance of this pathogen to this class of fungicides.

### Overview of the transcriptional responses of *M. perniciosa* to a strobilurin fungicide

**Figure 2:**
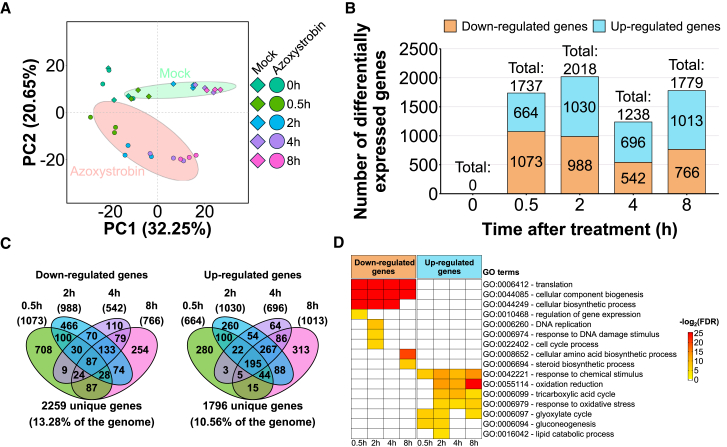
Overview of the transcriptional responses of *M. perniciosa* FA553 upon treatment with a strobilurin fungicide (A) Principal component analysis (PCA) shows that the transcriptional profiles of mock and azoxystrobin-treated mycelia are clearly distinct at all time points, except for T = 0 h (samples harvested immediately after the start of the experiment). Ellipses show the parametric smallest area around the mean that contains 70% of the probability mass for each group. (B) Number of differentially expressed genes at each time point of the experiment (0 h, 0.5 h, 2 h, 4 h, and 8 h). Here, azoxystrobin-treated mycelia were compared to their corresponding mock-treated controls. (C) Venn diagrams demonstrating the overlap of DEGs across different time points (left panel: down regulated genes, right panel: up regulated genes). (D) Gene ontology (GO) enrichment analysis revealed the upregulation of catabolic processes and energy metabolism pathways and downregulation of biological processes related to cellular multiplication, DNA replication and protein synthesis at most time points. The complete GO enrichment analysis and the corresponding statistics are provided in Table S2. See also Figure S3.

To investigate the molecular responses associated with strobilurin tolerance in *M. perniciosa*, we performed a genome-wide gene expression analysis in a time-course experiment spanning the first 8 h following azoxystrobin exposure (Figure S1). The rationale was to capture the early response of the fungus to the stress caused by the drug. For the *M. perniciosa* isolate FA553, 30 RNA-seq libraries were constructed from mock and azoxystrobin-treated mycelium at five time points (0 h, 0.5 h, 2 h, 4 h, and 8 h). A total of 382.82 million Illumina reads were generated (Figure S4A), with an average of 12.76 million reads per library (Figure S4B), most of which mapped to exons (Figure S4C). Principal Component Analysis (PCA) revealed a clear difference between the transcriptomes of control and azoxystrobin-treated mycelia (Figure 2A). Genes related to cellular detoxification and stress response contributed most to the separation between these two groups (described below).

Differentially expressed genes between mock and azoxystrobin-treated samples (false discovery rate [FDR] ≤ 0.01; fold-change of 1.5) were identified using edgeR.^19^ Of the 17,008 gene models in the *M. perniciosa* isolate FA553, 14,399 were considered expressed in our experiment (see STAR Methods for details). Among these, 3,969 were differentially expressed in at least one time-point of the experiment, representing 23% of the total *M. perniciosa* genes (Table S1). As expected, no genes were differentially expressed in samples harvested immediately after transferring the mycelium to azoxystrobin-amended culture medium (T = 0 h). The number of differentially expressed genes (DEGs) at subsequent time points varied from 1,238 (T = 4 h) to 2,018 (T = 2 h) (Figure 2B). Importantly, as little as 30 min of exposure to azoxystrobin was sufficient to affect the expression of thousands of genes. Overall, fungal responses to azoxystrobin at different time points were highly similar, with 1,660 genes (41.82% of DEGs) differentially expressed at more than one time point (Figure 2C; Table S1). Interestingly, gene ontology (GO) enrichment analysis revealed intense metabolic reprogramming, including upregulation of biological processes related to energy metabolism, such as cellular respiration, gluconeogenesis, and lipid catabolism (Figure 2D; Table S2). Conversely, biological processes associated with growth, such as DNA replication, translation, and cell cycle, were downregulated.

### The *M. perniciosa* respiratory chain is remodeled in response to azoxystrobin

**Figure 3:**
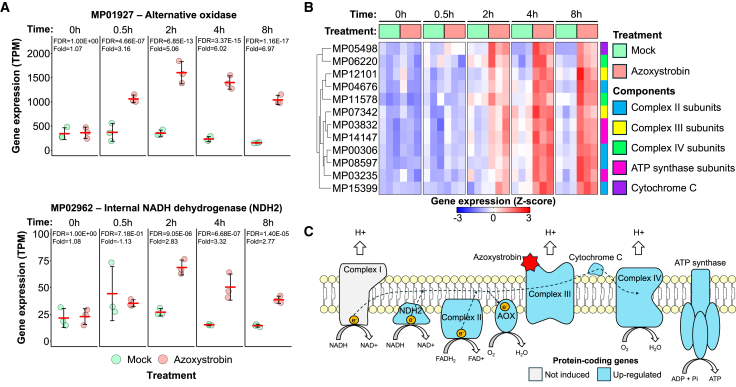
Azoxystrobin induces genes encoding components of the mitochondrial respiratory chain (A) Genes coding for an alternative oxidase (MP01927) and an internal NADH dehydrogenase (MP02962) are significantly upregulated upon azoxystrobin exposure. Error bars indicate the standard error (n= 3). (B) Expression pattern of genes coding for components of the main mitochondrial respiratory chain. Note the induction of components of most mitochondrial respiratory complexes, possibly as a compensatory response to alleviate the negative effects of azoxystrobin on ATP production. The complete list of differentially expressed genes associated with cellular respiration (including their functional annotation) is provided in Table S3. (C) Schematic representation of the mitochondrial respiratory chain, highlighting proteins whose corresponding genes were upregulated upon azoxystrobin treatment.

In agreement with our previous studies,^13^^,^^20^^,^^21^ *M. perniciosa* seems to mitigate the effects of mitochondrial complex III inhibition by the rapid upregulation of the *Mp*-*Aox* gene (Figure 3A), resulting in the activation of an alternative respiratory pathway that is insensitive to strobilurin. Indeed, AOX enzymatic activity has only been detected in *M. perniciosa* upon azoxystrobin exposure.^13^ Because this alternative pathway is less energy-efficient than the main respiratory chain,^22^ *M. perniciosa* cells likely experience ATP deprivation when exposed to azoxystrobin. Under these conditions, other enzymes and pathways may also play important roles to ensure fungal survival. A gene encoding a non-proton pumping internal NADH dehydrogenase (NDH-2) was differentially expressed 2 h after treatment (Figure 3A), possibly to alleviate azoxystrobin-induced oxidative stress. Additionally, at least 12 genes encoding components of the main mitochondrial respiratory chain (including subunits of complexes II, III and IV, and ATP synthase) were induced by azoxystrobin (Figure 3B; Table S3), likely as an attempt to compensate for the impaired cellular respiration. These results suggest that azoxystrobin treatment might cause the rearrangement and assembly of additional respiratory complexes (Table S3) to compensate for the decreased energy production caused by complex III inhibition.

### Azoxystrobin induces fatty acid and amino acid catabolism and the TCA cycle

In the presence of azoxystrobin, *M. perniciosa* seems to activate the catabolism of lipids to boost the supply of reduced coenzymes for the respiratory chain (Table S3). GO enrichment analysis revealed that ‘lipid catabolic processes’ (GO:0016042) were activated after 2 h of fungal exposure to azoxystrobin (Figure 2D). Indeed, a total of 18 genes involved in fatty acid catabolism were differentially expressed in the experiment (Table S3). Six genes encoding lipases, which catalyze the hydrolysis of triacylglycerols into free fatty acids and glycerol, were upregulated by azoxystrobin (Table S3). Furthermore, genes required for fatty acid activation (acyl-CoA synthase and acyl-carnitine transferase) were also strongly activated in azoxystrobin-treated mycelia (Table S3). Consistent with the use of fatty acids as an energy source, genes encoding three mitochondrial enzymes involved in β-oxidation (enoyl-CoA hydratase, 3-hydroxyacyl-CoA dehydrogenase, and 3-ketoacyl-CoA thiolase) were upregulated in response to the fungicide (Table S3). Notably, β-oxidation of fatty acids also occurs in peroxisomes.^23^ Accordingly, genes encoding the peroxisomal enzymes acyl-CoA oxidase, hydratase-dehydrogenase-epimerase and 3-ketoacyl thiolase, were also induced (Table S3). Thus, both peroxisomal and mitochondrial β-oxidation pathways were activated in response to azoxystrobin.

**Figure 4:**
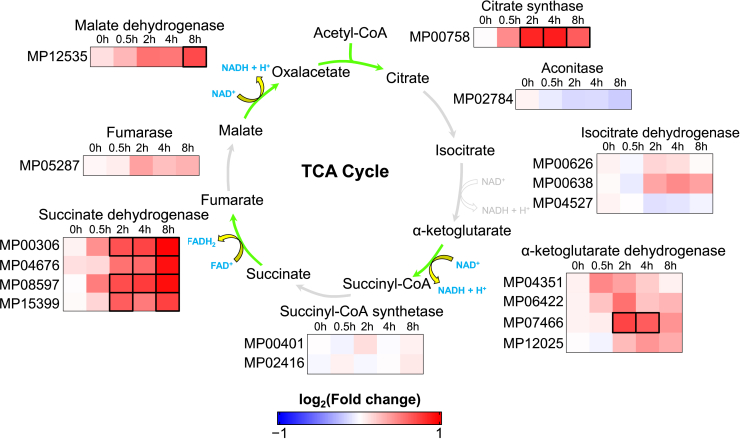
Key components of the TCA cycle are induced in azoxystrobin-treated mycelia The expression pattern of genes encoding enzymes of the TCA cycle are shown. Green arrows indicate azoxystrobin-induced reactions, while the reduced coenzymes produced in these reactions are highlighted in blue. Differentially expressed genes at specific time points are indicated by black bold borders. The complete gene expression data and differential expression results are provided in Table S3.

Reduced coenzymes (NADH and FADH_2_) and acetyl-CoA are the final products of β-oxidation. Both reduced coenzymes can serve as electron donors in the mitochondrial electron transport chain for ATP production.^24^ Moreover, acetyl-CoA can enter the tricarboxylic acid cycle (TCA) (Figure 4), generating even more NADH, FADH_2_ and ATP.^25^ The degradation of branched-chain amino acids (valine, leucine and isoleucine) also contributes to ATP production by providing the TCA cycle with acetyl-CoA.^26^ Notably, 9 genes encoding enzymes required for the catabolism of branched-chain amino acids were induced by azoxystrobin (Table S3). Interestingly, the GO term ‘Tricarboxylic Acid Cycle’ (GO:0006099) was enriched in the set of genes upregulated by the fungicide (Figure 2D). In particular, genes encoding three of the enzymes that catalyze the steps of the TCA cycle where the coenzymes NAD^+^ and FAD^+^ are reduced (i.e., α-ketoglutarate dehydrogenase, malate dehydrogenase and succinate dehydrogenase) were all activated in azoxystrobin-treated mycelia (Figure 4). These findings are consistent with the hypothesis that lipids and amino acids are catabolized to sustain cellular respiratory capacity and energy production upon azoxystrobin treatment, thus contributing to fungal survival.

### The glyoxylate cycle and gluconeogenesis are activated by azoxystrobin

In fungi, fatty acids can also be converted into glucose and precursors of the TCA cycle via the glyoxylate cycle.^27^ This process occurs in glyoxysomes, which are specialized peroxisomes that contain the enzymes isocitrate lyase and malate synthase, both exclusive of the glyoxylate cycle.^28^ Remarkably, genes encoding these enzymes are induced at three (isocitrate lyase) and four (malate synthase) different time points (Table S3). Isocitrate lyase cleaves isocitrate into succinate and glyoxylate. Glyoxylate is then condensed with acetyl-CoA by malate synthase, producing malate, whereas succinate is transported to the mitochondrial matrix through a dicarboxylate transporter.^29^ In agreement, a mitochondrial dicarboxylate transporter is activated after 2 h of azoxystrobin treatment (Table S3). Succinate then enters the TCA cycle, where it is converted into malate, which is transported to the cytosol and converted into glucose via enzymes involved in gluconeogenesis, such as PEPCK (Phosphoenolpyruvate carboxykinase), fructose 1,6-bisphosphatase and glucose 6-phosphatase. Except for glucose 6-phosphatase, which catalyzes the conversion of glucose 6-phosphate to glucose, genes encoding these enzymes were activated at all four time points (Table S3). These results indicate that both the glyoxylate cycle and gluconeogenesis pathways are induced in response to azoxystrobin.

### Fitness costs associated with azoxystrobin exposure

*M. perniciosa* shows reduced growth in the presence of azoxystrobin (Figures 1 and S2). In agreement, biological processes associated with cell growth and proliferation were repressed in response to the fungicide (Figure 2D). Genes with GO annotation for cell cycle (GO:0022402), DNA replication (GO:0006260), gene expression (GO:0010468), translation (GO:0006412) and steroid biosynthesis (GO:0006694) were repressed upon azoxystrobin treatment (Figure 2D). A total of 358 genes related to these processes were downregulated in, at least, one time point of the experiment (Table S4). These results suggest that basic biological processes are suppressed in response to energy-depriving conditions triggered by azoxystrobin.

#### Cell cycle progression and DNA replication

A set of 29 genes required for cell cycle progression was repressed by azoxystrobin treatment (Table S4), including those encoding the cdk2-cdc13 complex and the M-phase inducer phosphatase, which are essential for cells to enter mitosis.^30^^,^^31^ In addition, 46 genes coding for DNA primases, polymerases, ligases, and components of the pre-replication complex, which are important to initiate DNA replication,^32^ were also repressed, mostly, at the 2 h-time point (Table S4). Remarkably, we verified downregulation of 13 genes associated with DNA repair, such as the DNA polymerase β, RAD52 and PCNA^33^^,^^34^^,^^35^ (Table S4). Collectively, these results suggest that both the cell cycle and DNA replication/repair processes are impaired in response to azoxystrobin.

#### mRNA and protein synthesis

A total of 61 genes coding for components of the transcriptional machinery were repressed in response to azoxystrobin in at least one time point (Table S4). These include the gene coding for Rpa49, a subunit of RNA polymerase I^36^ and CDC73 (Cell Division Control 73), which is a constituent of the PAF1 complex, a key regulator of mRNA synthesis.^37^ Furthermore, 193 genes coding for translation-related proteins were mostly repressed 30 min after azoxystrobin exposure (Table S4). They include 80 genes encoding components of the 40S and 60S ribosomal subunits and 18 genes coding for tRNA synthetases. These results indicate that both the transcription and translation machineries of *M. perniciosa* are negatively affected by azoxystrobin treatment.

#### Ergosterol biosynthesis

Of the 23 genes putatively involved in ergosterol biosynthesis,^38^ 16 were downregulated in mycelia exposed to azoxystrobin in at least one time point (Table S4). These include genes encoding the enzymes hydroxymethylglutaryl-CoA synthase, lanosterol 14-α-demethylase and δ-sterol reductase (Table S4), all of which are necessary for ergosterol biosynthesis. Notably, ergosterol is a major component of fungal cell membranes, playing a role in maintaining membrane fluidity and integrity.^38^ Our results suggest that azoxystrobin interferes with ergosterol biosynthesis, potentially compromising membrane integrity and impairing fungal growth.

### *M. perniciosa* activates a variety of detoxification mechanisms in response to azoxystrobin

**Figure 5:**
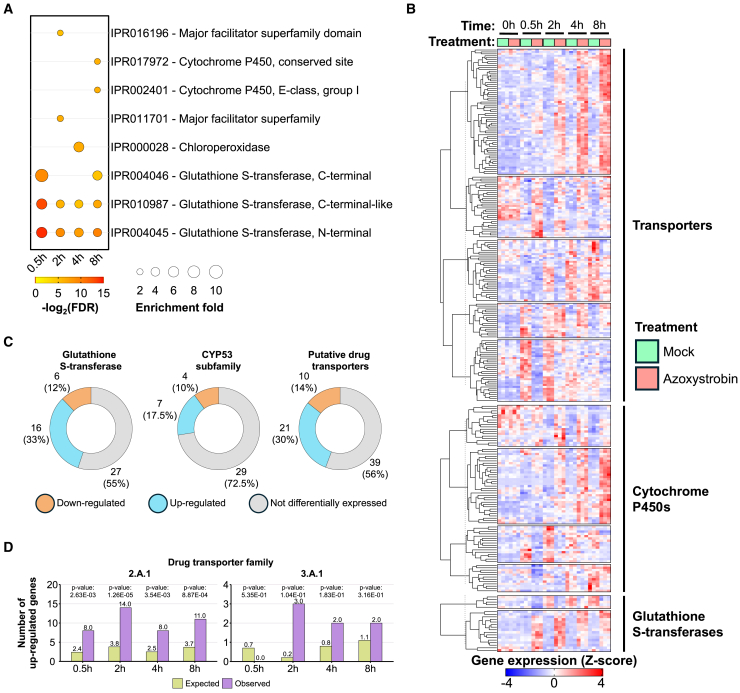
Activation of detoxification mechanisms in *M. perniciosa* in response to azoxystrobin (A) InterPro enrichment analysis showing overrepresented terms in the azoxystrobin-treated mycelium (FDR < 0.05). (B) Hierarchical clustering of genes encoding detoxification factors that are differentially expressed upon treatment with azoxystrobin. (C) Number of detoxification factors upregulated, downregulated, or not differentially expressed in the presence of azoxystrobin. (D) Enrichment analysis of candidate drug transporters in the 2.A.1 and 3.A.1 superfamilies (MFS and ABC transporters, respectively). Members of the 2.A.1 superfamily are more prevalent among the set of upregulated genes than the expected by chance. Genes encoding members of the 3.A.1 superfamily are activated by azoxystrobin, but the enrichment of this superfamily is not statistically significant.

In addition to the metabolic rearrangement observed in *M. perniciosa* in response to azoxystrobin, the analysis of the fungal transcriptome suggested the activation of diverse detoxification mechanisms in the azoxystrobin-treated mycelium (Figure 5A; Table S5). These mechanisms may independently function to modify, degrade, or excrete the fungicide from fungal cells, alleviating its toxic effects. Moreover, genes associated with the mitigation of oxidative stress were also upregulated in the azoxystrobin-treated mycelium.

#### Glutathione S-transferase (GST)

GSTs play a central role in drug resistance and detoxification pathways. These enzymes catalyze the conjugation of glutathione to toxic endogenous or xenobiotic substrates, preventing these molecules from reacting with essential cellular macromolecules.^39^ The *M. perniciosa* genome encodes 52 GST genes, of which 15 (29%) were induced and only 6 (11%) were repressed by azoxystrobin, suggesting a role for glutathione metabolism in the *M. perniciosa* tolerance to strobilurin fungicides (Figures 5B and 5C; Table S5).

#### Cytochrome P450

Members of the cytochrome P450 (CYP450) family were also differentially expressed in the presence of azoxystrobin. CYP450 enzymes can catalyze the conversion of xenobiotic compounds into easily excreted, less toxic, products. Interestingly, the CYP450 family is expanded in the *M. perniciosa* genome,^14^ suggesting a high potential for detoxification in this fungus. Of the 326 CYP450 genes in *M. perniciosa*, 79 (24%) were differentially expressed (52 upregulated, 27 downregulated) (Figure 5B; Table S5). In addition, 23 genes coding for CYP450s in *M. perniciosa* belong to the CYP53 subfamily, which is involved in the detoxification of benzoate, an intermediate in the degradation of aromatic compounds in fungi.^40^ Interestingly, five of these genes were induced by azoxystrobin (Figure 5C; Table S5), which is an aromatic compound, suggesting the participation of CYP53 in azoxystrobin degradation.

#### Efflux pumps

At least 179 genes encoding members of different families of transporters were differentially expressed in response to azoxystrobin. The *M. perniciosa* genome encodes 1,413 putative transporters, including 258 from the 2.A.1 superfamily (Major Facilitator Superfamily - MFS) and 56 from the 3.A.1 superfamily (ATP-binding cassette - ABC) (Figures 5D and 5E; Table S5). These transporters likely function as efflux pumps, transporting xenobiotic compounds by transporting them to the extracellular space and preventing their lethal accumulation.^41^ Within these subfamilies, at least 70 transporters were classified as putative drug transporters, and 21 of them (17 of the 2.A.1 and 4 of the 3.A.1 superfamily) were upregulated by azoxystrobin (Figures 5D and 5E; Table S5). These findings suggest that *M. perniciosa* uses membrane transporters to pump azoxystrobin to the extracellular space, thus preventing its toxic accumulation in the fungal cell.

### Exposure of *M. perniciosa* to azoxystrobin promoted the emergence of a resistant mutant

**Figure 6:**
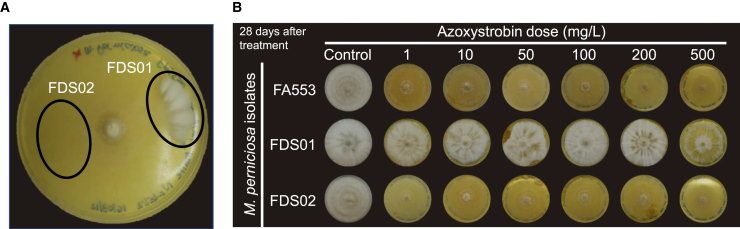
Isolation of an *M. perniciosa* mutant with reduced sensitivity to azoxystrobin (A) In the presence of azoxystrobin, *M. perniciosa* grows as thin mycelia on solid medium. Interestingly, a mycelial sector with an increased growth rate was observed, indicating the emergence of a phenotype with enhanced resistance to the fungicide. This mycelial sector was isolated and named FDS01. As a control, a sector displaying wildtype morphology was also isolated and named FDS02. The image was taken 41 days after inoculation. (B) The isolates FDS01, FDS02, and FA553 (the parental isolate) were cultivated in media with increasing concentrations of azoxystrobin. All three isolates display similar morphology in MYEA medium. However, while the isolates FA553 and FDS02 grow as thin mycelia in the presence of azoxystrobin, the FDS01 isolate shows vigorous growth, even at higher fungicide concentrations. Images were taken 28 days after inoculation.

The transcriptional analysis of *M. perniciosa* in response to azoxystrobin revealed several molecular alterations that may enable fungal survival in the presence of this fungicide, albeit at the cost of reduced growth. Remarkably, during the cultivation of *M. perniciosa* isolate FA553 on solid medium containing azoxystrobin (200 mg/L), we noticed the development of a morphologically distinct mycelial sector that grew faster than the rest of the mycelium on the same plate (Figure 6A). This presumably resistant mycelial sector was isolated, replicated *in vitro* and named FDS01 (FA553-derived sector 01). Another sector from the same Petri dish, displaying wildtype-like morphology, was also isolated and named FDS02 (FA553-derived sector 02). The three isolates (i.e., FA553, FDS01 and FDS02) were subsequently cultivated in the presence of azoxystrobin to evaluate their sensitivity to this fungicide (Figure 6B). As expected, the growth of the FA553 and FDS02 isolates was inhibited, but not entirely blocked by azoxystrobin. Remarkably, FDS01 showed vigorous growth even at high concentrations of azoxystrobin (i.e., 500 mg/L), suggesting that this isolate is resistant to the fungicide (Figure 6B).

As described above, single-point mutations in the *CytB* gene can confer resistance to strobilurin fungicides.^11^ These mutations, such as F129L and G143A, alter the binding of strobilurins to cytochrome *b*, abolishing their fungicidal effect. However, no such mutation in the cytochrome *b* gene was detected in the FDS01 mutant (Figure S3). Additionally, although AOX has already been shown to mitigate azoxystrobin effects on *M. perniciosa*,^13^ *Mp*-*Aox* is similarly upregulated in all three isolates along the time-course experiment (Figure S5). This suggests that the azoxystrobin-resistant phenotype of the FDS01 isolate is unlikely to be due to differences in Mp-AOX activity levels. These results indicate that well-described strobilurin resistance mechanisms (i.e., mutations in the *CytB* gene and Mp-AOX activity) do not account for the increased tolerance of the FDS01 isolate to azoxystrobin, implying the involvement of additional mechanisms.

### The FDS01 isolate shows intense transcription deregulation

The RNA-seq results described above focus on the FA553 isolate, highlighting the global effects of azoxystrobin on the wildtype mycelia of *M. perniciosa*. To investigate possible transcriptional changes linked to the azoxystrobin resistance phenotype of FDS01, RNA-seq experiments were also conducted on the FDS01 and FDS02 isolates. Along with the 30 RNA-seq libraries for FA553, 60 additional libraries were generated for FDS01 and FDS02 (30 for each isolate; Figure S4). These libraries correspond to biological triplicates of mock and azoxystrobin-treated samples at five time points (i.e., 0 h, 0.5 h, 2 h, 4 h, and 8 h).

**Figure 7:**
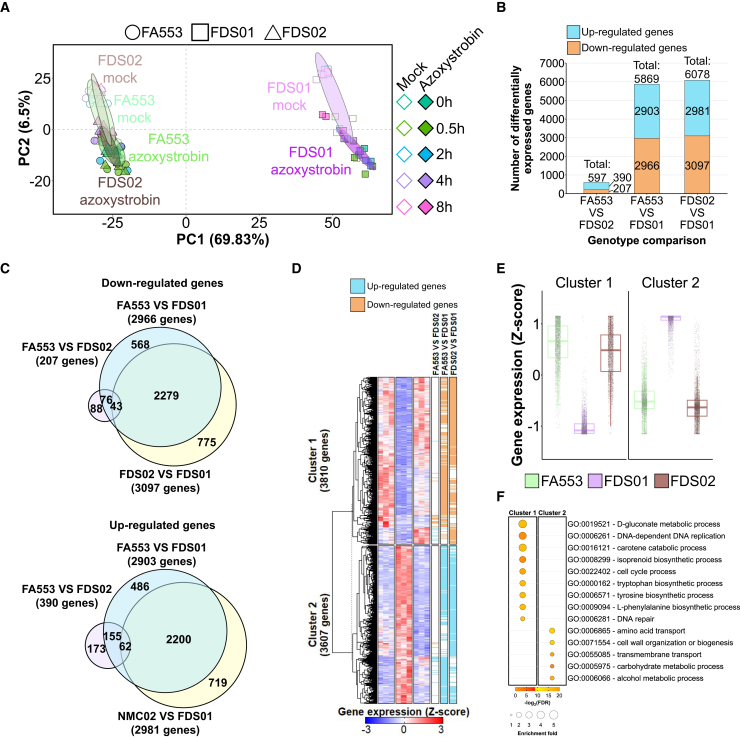
The mutant strain FDS01 shows an altered transcriptional program (A) Principal Component Analysis (PCA) plot based on the transcriptomes of the isolates FA553, FDS01, and FDS02. The FDS01 transcriptional profile is remarkably different from the transcriptomes of the other two isolates, regardless of the presence of azoxystrobin. The color code represents different time points in the experiment. Empty diamonds represent control samples, while filled diamonds represent azoxystrobin-treated samples of the three isolates. Circles, squares, and triangles represent triplicates from FA553, FDS01, and FDS02, respectively. Ellipses show the parametric smallest area around the mean that contains 60% of the probability mass for each group. (B) Number of downregulated (orange) and upregulated (blue) genes between genotypes. (C) Overlap of downregulated (top) and upregulated genes (bottom) based on the comparison of the genotypes at time point T = 0 h. (D) Hierarchical clustering of the 7417 differentially expressed genes among the genotypes FA553, FDS01, and FDS02. The FDS01 mutant displays a distinct transcriptional signature compared to the FA553 and FDS02 wildtype isolates. (E) Expression patterns for clusters 1 and 2, as defined in the heatmap shown in panel D. (F) Gene Ontology (GO) enrichment analysis indicates the enriched biological processes in each cluster. A detailed GO enrichment analysis, including the corresponding statistics, can be found in Table S7. See also Figures S3–S5.

A Principal Component Analysis (PCA) revealed that the parental strain FA553 and its wildtype derivative FDS02 share a highly similar transcriptional signature (Figure 7A). This result validates the key conclusions regarding the effects of azoxystrobin on *M. perniciosa* using a second isolate (FDS02). Conversely, the azoxystrobin-resistant mutant FDS01 was clearly different from the other two isolates, regardless of the presence of azoxystrobin. Yet, the fungicide had a pronounced effect on the transcriptomes of all three genotypes throughout the time course (Figure 7A), supporting the observation that azoxystrobin triggers extensive transcriptional reprogramming of fungal metabolism.

To further explore the differences between FDS01 and the other two isolates, we performed pairwise comparisons in the absence of azoxystrobin at time point T = 0 h. A total of 7,417 genes were differentially expressed (FDR ≤ 0.01 and Fold-change ≥ 1.5) across the three genotypes (Table S6). The FDS01 mutant showed 5,869 and 6,078 differentially expressed genes compared to FA553 and FDS02, respectively (Figures 7B and 7C), whereas only 597 genes were differentially expressed between FA553 and FDS02. These results confirm the high similarity between FA553 and FDS02, while underscoring the distinct nature of the FDS01 mutant.

Hierarchical clustering of the differentially expressed genes among genotypes revealed two distinct clusters (Figure 7D). Cluster 1 consists of genes that were more highly expressed in the wildtype genotypes (FA553 and FDS02) but weakly expressed in the FDS01 mutant (Figures 7D and 7E). Conversely, cluster 2 contains genes with higher expression in FDS01 and lower expression levels in the other two isolates. Notably, cluster 1 includes genes involved in basic biological processes, such as DNA replication, cell cycle and amino acid biosynthesis, while cluster 2 is enriched for genes associated with metabolic process, transmembrane transport, and cell wall biogenesis (Figure 7F; Table S7).

### Putative growth and transcriptional regulators are mutated in FDS01

**Figure 8:**
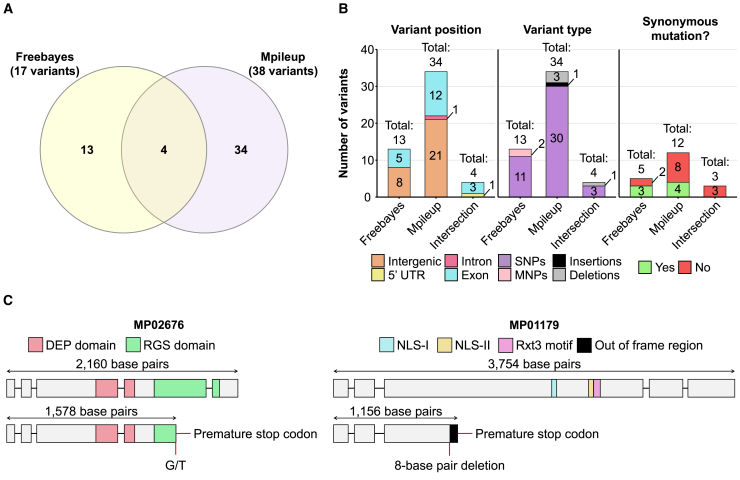
Identification of potential genomic variants associated with the FDS01 mutant phenotype (A) Venn diagram showing the overlap of variants found by Freebayes and Mpileup when comparing FA553 and FDS01 genomes. (B) Barplots indicating the number of variants uniquely identified by Freebayes, Mpileup and their intersection. The variants were classified by their position in *M. perniciosa* genome, their type and whether they encode an identical protein in both FA553 and FDS01 genomes (i.e., result in synonymous mutations; only applicable for SNPs in exons). (C) The MP02676 wildtype allele from FA553 and FDS02 isolates encodes a putative regulator of G protein signaling that possesses a DEP and an RGS domain. In the FDS01 genome, an SNP (G to T) results in a premature stop codon, producing a truncated protein with partial loss of the RGS domain, possibly affecting its function. The MP01179 wildtype allele encodes a putative transcriptional regulator containing two nuclear localization signals (NLS-I and NLS-II) and an Rxt3 motif. An 8-base pair deletion in the FDS01 genome results in a frameshift, leading to a premature stop-codon and a truncated protein. This truncation results in loss of both NLSs and the Rxt3 motif, likely compromising protein function. Exons are indicated by gray boxes, while introns are shown as lines. Figure S6 shows these two mutations in reads derived from the FDS01 genome.

A genomic survey was conducted to identify differences in FDS01 compared to the wildtype genotypes FA553 and FDS02. For this, genomic DNA from these isolates was shotgun-sequenced, and the resulting reads were aligned to the *M. perniciosa* reference assembly (isolate FA553). Four genomic differences were consistently identified by the two variant-calling methods employed (freebayes and Mpileup) in FDS01, but not in FA553 and FDS02 (Figures 8A and 8B; Table S8). Three of these variants were single nucleotide polymorphisms (SNPs) in protein-coding genes (MP02676, MP10135 and MP12249), whereas the remaining one was an 8-nucleotide deletion in MP01179.

Among the three high-confidence SNPs identified in FDS01, one was located in the 5′ UTR of MP12249 (which encodes a putative terpene synthase) and another resulted in a non-synonymous mutation in MP10135. This mutation replaces a glutamate with a lysine in the encoded protein, which has no assigned function. The third SNP introduced a premature stop codon in MP02676 (Figure 8C), a gene that encodes a putative regulator of G-proteins. G-proteins control numerous cellular processes in fungi, including sexual and vegetative development.^42^^,^^43^ The early stop codon causes partial loss of the RGS (regulator of G-protein signaling) domain, which is required for G-protein inactivation.^42^^,^^43^

Finally, the 8-nucleotide deletion in MP01179 (Figure 8C) also introduced a premature stop-codon, resulting in a truncated protein lacking 830 amino acids compared to its wildtype counterpart. and a motif (Figure 8C) shared with the yeast Rxt3 protein, a component of the transcriptional repressor complex Rpd3.^44^ Additionally, it shows 33.25% identity with a putative transcriptional repressor from *Coprinopsis cinerea* (GenBank: KAG2011931.1), and PSI-BLAST searches revealed 25% identity with the SAFB (Scaffold Attachment Factor Box)-Like transcriptional regulator from *Mus musculus*, which functions as a general transcriptional inhibitor.^45^ Thus, MP01179 likely encodes a novel transcriptional regulator that lost its function in the FDS01 mutant due to nucleotide deletions. This finding is consistent with the major transcriptional alterations observed in the FDS01 isolate (Figure 7).

## Discussion

WBD is a major limiting factor for cacao cultivation in the Americas. Previous attempts to control the disease using strobilurin fungicides have been unsuccessful.^13^ Here, we show that *M. perniciosa* can tolerate high concentrations of strobilurins *in vitro*, and this tolerance is not explained by the typical mutations described in the *Cytb* gene (Figure S3). Our results indicate that, in the presence of strobilurin, *M. perniciosa* undergoes intense metabolic reprogramming, including the repression of basic biological processes and activation of a series of catabolic processes and detoxification mechanisms. Importantly, *M. perniciosa* remains recalcitrant to genetic transformation, limiting our ability to confirm the molecular mechanisms required for strobilurin resistance through knockout mutants or overexpression lines. Nonetheless, the candidate genes identified in this study represent a valuable foundation for future functional studies as the necessary tools become available.

Strobilurins inhibit complex III of the main respiratory chain, leading to ATP deprivation and oxidative stress, which can eventually cause fungal death.^46^ The alternative oxidase enzyme, found in many phytopathogenic fungi,^47^ offers a bypass to complexes III and IV, alleviating the negative effects of strobilurin.^48^ Indeed, the *M. perniciosa* AOX is important for strobilurin tolerance.^13^^,^^20^^,^^21^ Accordingly, we verified a rapid upregulation of *Mp*-*Aox* upon azoxystrobin treatment (Figure 3A). In addition, an external NADH dehydrogenase gene (*NDH-2*) was induced by the fungicide (Figure 3A). Alternative non-proton pumping NADH dehydrogenases (e.g., NDH-2) are known to mitigate oxidative stress by promoting NADH oxidation, thus enhancing electron transport and metabolic flux.^49^^,^^50^ While AOX and NDH-2 activity might promote fungal survival by reducing the generation of oxidative stress, ATP production is still negatively impacted due to their non-proton pumping nature.^47^^,^^50^ Importantly, these transcriptional alterations agree with the observed reduced growth of *M. perniciosa* mycelia in the presence of strobilurin (Figure 1).

To overcome azoxystrobin-induced ATP deprivation, *M. perniciosa* seems to activate important catabolic pathways. Genes encoding lipases and enzymes involved in both mitochondrial and peroxisomal β-oxidation were strongly upregulated in response to azoxystrobin, suggesting activation of lipid degradation (Table S3). Interestingly, both pathways play important roles in the survival and pathogenicity of several fungi.^27^^,^^51^^,^^52^^,^^53^ NADH, FADH_2_ and acetyl-CoA are products of the β-oxidation pathway. While NADH and FADH_2_ can be directly used in the electron transport chain to generate ATP, acetyl-CoA is oxidized in the TCA cycle to produce more reduced coenzymes and ATP.^54^ Consistent with this, genes coding for enzymes of the TCA cycle were also induced (Figure 4). Besides lipid catabolism, azoxystrobin appears to induce the degradation of branched-chain amino acids, which might generate additional acetyl-CoA for the TCA cycle, further contributing to ATP production (Table S3).^26^ These results suggest that *M. perniciosa* uses alternative energy sources to compensate for azoxystrobin-triggered ATP deprivation.

Genes encoding key enzymes of the glyoxylate cycle, isocitrate lyase (ICL) and malate synthase (MLS), were induced in *M. perniciosa* upon azoxystrobin treatment (Table S3). These enzymes are exclusive to the glyoxylate cycle and generate intermediaries for the TCA cycle.^55^ Previous studies have described that these glyoxysomal enzymes play crucial roles on survival and pathogenicity of both plant and human pathogenic fungi. In *Candida albicans*, for example, the glyoxylate cycle enables yeast growth in nutrient-limited environments, within phagocytic cells.^56^ Based on these findings, compounds with potential inhibitory effects on ICL have been proposed as drug targets for *C. albicans*.^57^ Likewise, azoxystrobin treatment triggers an energy-limiting condition in *M. perniciosa*, which results in the activation of the glyoxylate cycle. Therefore, our results suggest that the enzymes ICL and MLS might be prospective drug targets to be explored, potentially providing new options of drugs to be combined with azoxystrobin for the control of fungal diseases.

Even when exposed to high doses of azoxystrobin, *M. perniciosa* seems to produce enough energy to survive, albeit its growth rate is significantly reduced (Figure 1). This suggests that metabolic trade-offs may occur under fungicide exposure, favoring catabolism over anabolism for ATP generation. For instance, DNA, RNA and protein synthesis are primary consumers of ATP in a cell.^58^ In *M. perniciosa*, genes involved in cell cycle progression, DNA replication, transcription and translation were downregulated in the azoxystrobin-treated mycelia (Table S4). Additionally, fungal genes required for ergosterol biosynthesis were repressed in response to the fungicide (Table S4). Ergosterol is a major component of fungal cell membranes and is essential for maintaining membrane structure, fluidity and permeability.^59^ Therefore, inhibition of ergosterol biosynthesis negatively impacts fungal growth.^60^ For example, in *Fusarium graminearum*, the inhibition of ergosterol biosynthesis by thymol results in reduced growth rate.^61^ Similarly, azole antifungals target the 14α-demethylase enzyme (CYP51) in the ergosterol biosynthesis pathway, effectively inhibiting fungal growth.^62^ In *M. perniciosa*, the possible repression of ergosterol biosynthesis by azoxystrobin (Table S4) could compromise cell membrane integrity, contributing to the observed reduction in fungal growth.

*M. perniciosa* seems to activate a variety of detoxification mechanisms in response to azoxystrobin. Cytochrome P450 proteins are involved in a large diversity of biological processes, including degradation of xenobiotic compounds. Specifically, enzymes of the CYP53 subfamily are essential for the degradation of aromatic compounds, as they catalyze the para-hydroxylation of benzoate, a toxic intermediate.^63^ Our transcriptomic data showed that members of the CYP53 family are upregulated by azoxystrobin (Figure 5C), which is an aromatic molecule. This suggests that the activation of these enzymes may be an attempt to detoxify the strobilurin fungicide. Notably, CYP53 family members are highly conserved in both ascomycetes and basidiomycetes and are critical for *Aspergillus nidulans* survival in the presence of benzoate.^64^ Furthermore, no homologs of this family is present in higher eucaryotes,^63^ making these enzymes potential drug targets. Drug transporters may also contribute to *M. perniciosa* detoxification response to azoxystrobin. In fungi, transporters can function as efflux pumps, expelling xenobiotic compounds into the external environment, thereby preventing their toxic accumulation inside the cells and contributing to pathogen insensitivity or resistance to fungicides.^65^ The major superfamilies of drug transporters, 2.A.1 (MFS) and 3.A.1 (ABC), are often associated with multi-drug resistance in phytopathogenic fungi.^8^ Genes encoding proteins from both superfamilies were activated in the *M. perniciosa* mycelia treated with azoxystrobin (Figure 5D). Additionally, a total of 15 genes encoding glutathione S-transferases (GSTs) were upregulated in response to azoxystrobin (Figure 5C). These enzymes are involved in the detoxification of xenobiotic compounds and protection against oxidative stress.^66^ Moreover, GSTs have been linked to resistance against the fungicides chlorothalonil and benzimidazole.^67^^,^^68^ Therefore, our results suggest that *M. perniciosa* may also employ GSTs to mitigate the cytotoxic effects of azoxystrobin (Figure 5C).

The indiscriminate use of fungicides can drive the emergence of resistant fungal strains. The lack of rotation in fungicide application and prolonged use of chemicals have contributed to the emergence of resistant isolates in several fungal pathogens.^69^ Antimicrobials often expose pathogens to DNA damage and mutagenesis,^70^^,^^71^^,^^72^ although these effects can be usually mitigated by cellular DNA repair systems.^73^ However, deregulation of these protective systems can lead to increased genomic instability and the development of drug resistance.^74^^,^^75^ Interestingly, azoxystrobin treatment downregulated genes related to DNA repair (Table S4), potentially increasing genetic diversification and facilitating the emergence of fungicide resistance. Indeed, after long-term exposure of *M. perniciosa* to azoxystrobin under laboratory conditions, we isolated a mycelial sector displaying vigorous growth (named FDS01), suggesting the spontaneous emergence of a resistant mutant (Figure 6). Unlike the parental strain, which tolerates strobilurins but shows reduced growth in the presence of azoxystrobin, growth of the FDS01 mutant was unaffected. Although single-point mutations in the *CytB* gene are typically responsible for resistance to strobilurin fungicides,^11^ no such mutations were found in FDS01 (Figure S3). Furthermore, AOX activity, known to enhance strobilurin tolerance,^13^ did not differ in expression between the resistant mutant and wildtype isolates FA553 and FDS02 (Figure S5). These findings suggest that FDS01 resistance to strobilurins arises from novel, non-canonical mechanisms, distinct from known *CytB* mutations or AOX-mediated pathway. In fungi, drug resistance mechanisms can involve mutations or altered expression of specific genes, epigenetic modifications, or even horizontal gene transfer (HGT).^76^^,^^77^^,^^78^

Genome analysis of FDS01 revealed an 8-nucleotide deletion in a putative transcription factor (MP01179 gene), as well as a non-synonymous mutation in an RGS-like protein (MP02676 gene), both of which could explain the mutant phenotype (Figure 8). The deletion in the gene MP01179 produces a premature stop codon resulting in a truncated protein with only 335 of the 1165 amino acids present in the wildtype protein. This truncated version lacks both the two predicted NLSs (nuclear localization signals) which likely prevents the protein from entering the nucleus, thus abolishing its function as a transcriptional regulator.^79^ Indeed, transcriptional regulation plays an important role in the development of fungicide resistance. A previously described example is the transcription factor sreA from *Penicillium digitatum*, which can contribute to resistance to the azole fungicide prochloraz by regulating the expression of *erg11*, a gene encoding a protein targeted by the fungicide.^80^ Likewise, fungal zinc-cluster transcription factors can promote multidrug resistance by upregulating genes that encode efflux pumps, which actively export toxic compounds out of the cell.^81^ Since transcription factors control gene expression, mutations in these regulators can disrupt their normal function and further promote resistance. For instance, a gain-of-function mutation in the transcription factor XDR1 from *Sclerotinia homoeocarpa* resulted in multidrug resistance through the overexpression of genes involved in xenobiotic detoxification.^82^ Similarly, mutations in MP01179 may contribute to the altered transcriptional program of FDS01 and its enhanced resistance, further pointing to the pivotal role of transcriptional regulation in the development of fungicide resistance.^80^^,^^81^^,^^82^

The MP02676 gene encodes a protein containing DEP and RGS domains, which are typical of repressors of G-protein signaling,^83^^,^^84^ a class of molecules reported to have distinct roles in fungi.^42^^,^^43^ These repressors use their RGS domain to deactivate G-proteins.^42^^,^^43^ Interestingly, the SNP found in MP02676 produces an early stop codon, leading to the partial loss of the RGS domain (Figure 8C), which may compromise its function. In *A. nidulans* and *A. fumigatus*, the FlbA RGS has a major role in growth regulation, and its deletion results in increased hyphal growth.^85^^,^^86^^,^^87^ Although MP02676 shares only 34.55% similarity with *A. nidulans* FlbA (Uniprot: P38093.1), the partial loss of the MP02676 RGS domain in FDS01 could similarly contribute to its vigorous hyphal growth in the presence of azoxystrobin (Figure 6).

The hypotheses regarding the contribution of the mutated versions of MP01179 and MP02676 to the mutant phenotype could be tested through genetic complementation of FDS01 with the wildtype allele or by generating independent mutants of each of these genes. However, an efficient genetic manipulation technique for *M. perniciosa* has not yet been developed. Thus, despite the available evidence, we are currently unable to confirm that the loss of function of MP01179 and MP02676 confers resistance to azoxystrobin. Yet, the results and functional evidence we provide here may guide future studies related to these genes in either *M. perniciosa* or other fungal species. Importantly, future advancements in genetic manipulation of *M. perniciosa* will allow for the confirmation of our hypotheses. Beyond genetic mutations, other mechanisms, such as epigenetic modifications and HGT, may also drive the evolution of fungicide resistance, particularly in response to environmental stressors. While our current work does not explore these mechanisms, they represent promising directions for future functional and genomic studies aimed at determining whether epigenetic changes or HGT events underlie the FDS01 resistance phenotype observed in *M. perniciosa*.

In conclusion, our results suggest that *M. perniciosa* shifts its metabolism toward an energy-saving state and activates distinct detoxification processes to cope with azoxystrobin toxicity. Although this metabolic adjustment reduces the fungal growth rate, it enables survival and may favor the emergence of resistant strains. This study underscores the importance of combining molecules with different modes of action for effective pathogen control. In addition, designing hybrid bifunctional compounds that target independent molecules or pathways in the pathogen might also offer a promising strategy to improve fungicide efficiency and durability in agriculture.^88^ In this context, combining azoxystrobin with fungicides such as triazoles (affecting ergosterol biosynthesis) or carboxamides (affecting drug transporters), represent a practical application of this approach, potentially enhancing efficacy and limiting resistance development.^63^^,^^89^^,^^90^ Future studies will investigate the precise contribution of the identified azoxystrobin-responsive genes to *M. perniciosa* tolerance to strobilurins, aiming to determine whether they are viable targets for the development of new fungicides.

### Limitations of the study

Strobilurins disrupt oxidative phosphorylation by inhibiting mitochondrial complex III.^9^ To investigate potential mechanisms underlying *M. perniciosa* resistance to azoxystrobin, we conducted *in vitro* assays using the complex medium MYEA supplemented with azoxystrobin. This medium was chosen to reflect the physiological conditions the fungus encounters during disease progression.^91^^,^^92^^,^^93^ However, this approach does not exclude the possibility that the fungus may also rely on fermentation as a short-term survival strategy. While assays using non-fermentable carbon sources can more directly assess mitochondrial function and uncover resistance mechanisms linked to the disruption of oxidative phosphorylation, rich media more accurately simulate the *in planta* nutritional environment and may reveal broader adaptive responses relevant to natural infection dynamics.

Although our transcriptomic analysis identified several candidate genes potentially involved in fungicide resistance, functional validation was not possible because of the current lack of efficient genetic manipulation tools for *M. perniciosa*. Future studies will be required to confirm the roles of these genes in azoxystrobin resistance as such tools become available. Additionally, our findings are based solely on *in vitro* assays. While these experiments provide foundational insights, *in planta* validation will be essential to confirm their biological relevance and to inform effective field-applicable strategies for managing fungicide resistance.

## Resource availability

### Lead contact

Requests for further information and resources should be directed to and will be fulfilled by the lead contact, Daniela Paula de Toledo Thomazella (dthomazella@usp.br).

### Materials availability

This study did not generate new unique reagents.

### Data and code availability

• The raw transcriptomic data supporting our findings has been made publicly available at the NCBI Gene Expression Omnibus (GEO) under accession number GEO: GSE281565.
• This paper does not report original code.
• Any additional information required to reanalyze the data reported in this paper is available from the lead contact upon request.

## Acknowledgments

This study was supported by the São Paulo Research Foundation (10.13039/501100001807FAPESP) through fellowships awarded to P.F.V.P., C.V.C.M., G.L.F., and D.P.T.T. (process numbers 20/04773-8, 14/00802-2, 14/06181-0, 13/05979-5, and 12/09136-0). B.A.P. was financed by the Coordenação de Aperfeiçoamento de Pessoal de Nível Superior – Brasil (10.13039/501100002322CAPES) – Finance Code 001. P.J.P.L.T received funds from the National Council for Scientific and Technological Development (10.13039/501100003593CNPq; grants 308349/2022-9 and 443671/2018-4), the 10.13039/501100013275Serrapilheira Institute (grant G-1811-25705), and 10.13039/501100001807FAPESP (grant 18/24432-0). We thank the University of North Carolina High Throughput Sequencing Facility for their support in providing sequencing services and training, as well as Natália Mincov Costa for her technical support and assistance in maintaining *M. perniciosa* cultures.

## Author contributions

P.J.P.L.T. and D.P.T.T. designed the research and supervised the project; P.F.V.P., C.V.C.M., and B.A.P. analyzed data; P.F.V.P. and G.L.F. performed the fungal resistance assays and RNA extraction for the RNA-seq experiments; P.F.V.P. prepared the RNA-seq library with supervision from P.J.P.L.T. and P.M.; P.J.P.L.T. and C.V.C.M. processed the RNA-seq data and identified the genomic variants in the *M. perniciosa* genome. P.J.P.L.T., P.M., and G.A.G.P. provided experimental materials; C.V.C.M., P.F.V.P., B.A.P., P.J.P.L.T., and D.P.T.T. wrote the article with inputs from all authors; P.F.V.P. and G.L.F. edited the article; All authors have read and approved the final version of the manuscript.

## Declaration of interests

The authors declare no competing interests.

## STAR★Methods

### Key resources table

**Table:** 

REAGENT or RESOURCE	SOURCE	IDENTIFIER
**Chemicals, peptides, and recombinant proteins**		

Azoxystrobin (AMISTAR® WG)	Syngenta, Switzerland	N/A
Metominostrobin		N/A
Picoxystrobin		N/A

**Critical commercial assays**		

RNeasy Plant Mini Kit	QIAGEN, Germany	Cat#74904
TruSeq mRNA Stranded Sample Prep Kit	Illumina, USA	
Lab Chip RNA high sensitivity assay	Caliper, USA	
Qubit DNA BR assay kit	QIAGEN, Germany	
TruSeq DNA Nano Kit	Illumina, USA	

**Deposited data**		

RNA-seq data	This paper	GEO: GSE281565

**Experimental models: Organisms/strains**		

*Moniliophthora perniciosa* isolate FA553	Gonçalo Pereira, State University of Campinas	N/A
*M. perniciosa* isolate FDS01	Gonçalo Pereira, State University of Campinas	N/A
*M. perniciosa* isolate FDS02	Gonçalo Pereira, State University of Campinas	N/A
*M. perniciosa* isolate FPN1	Gonçalo Pereira, State University of Campinas	N/A
*M. perniciosa* isolate CP02	Gonçalo Pereira, State University of Campinas	N/A
*M. perniciosa* isolate BP10	Gonçalo Pereira, State University of Campinas	N/A
*M. perniciosa* isolate Ilhéus B	Gonçalo Pereira, State University of Campinas	N/A
*M. perniciosa* isolate Ilhéus A	Gonçalo Pereira, State University of Campinas	N/A
*M. perniciosa* isolate WMA5	Gonçalo Pereira, State University of Campinas	N/A
*M. perniciosa* isolate STL1	Gonçalo Pereira, State University of Campinas	N/A
*M. perniciosa* isolate WMA5_B	Gonçalo Pereira, State University of Campinas	N/A
*M. perniciosa* isolate APS1	Gonçalo Pereira, State University of Campinas	N/A
*M. perniciosa* isolate 736.01	Gonçalo Pereira, State University of Campinas	N/A

**Software and algorithms**		

FASTX-Toolkit v0.0.13.2	Hannon 2010	https://github.com/agordon/fastx_toolkit
Cutadapt v1.3	Martin 2011	https://github.com/marcelm/cutadapt/
HISAT2 v2.1.0	Kim et al. 2019	https://daehwankimlab.github.io/hisat2/
HTSeq v0.6.1p1	Anders et al. 2015	https://github.com/simon-anders/htseq
edgeR package v3.18.1	Robinson et al. 2010	https://bioconductor.org/packages/release/bioc/html/edgeR.html
AMOR package v0.2-2		https://github.com/surh/AMOR
ComplexHeatmap package v2.16.0	Gu et al. 2016	https://bioconductor.org/packages/release/bioc/html/ComplexHeatmap.html
Cytoscape	Shannon et al. 2003	https://cytoscape.org/
FastQC v0.11.8	Babraham Bioinformatics, UK	https://github.com/s-andrews/FastQC
Trimmomatic v0.36	Bolger et al. 2014	http://www.usadellab.org/cms/?page=trimmomatic
Bowtie2 v2.4.0	Langmead and Salzberg 2012	https://github.com/BenLangmead/bowtie2
FreeBayes v1.1.0	Garrison and Marth 2012	https://github.com/freebayes/freebayes
BCFtools mpileup	Danecek et al. 2021	https://github.com/samtools/bcftools
Picard Tools v2.23.4	Broad Institute, USA	https://github.com/broadinstitute/picard
BCFtools isec v1.11		
VcfFilter v0.2		https://github.com/biopet/vcffilter
SnpSift v4.3t	Ruden et al. 2012	https://pcingola.github.io/SnpEff/

**Other**		

Sciclone G3 Automated Liquid Handling Workstation	PerkinElmer	
Illumina HiSeq 2500 platform	Illumina	Cat#SY-401-2501

### Experimental model and study participant details

The *M. perniciosa* isolates FA553, along with its derivatives FDS01 (FA553-derived sector 01; insensitive to azoxystrobin) and FDS02 (FA553-derived sector 02; parental phenotype) were used in this study. Fungal cultures were regularly maintained in Malt Yeast Extract Agar (MYEA) plates (17 g/L malt extract, 5 g/L yeast extract and 20 g/L agar) in an incubator at 28°C. Liquid cultures in Malt light medium (2 g/L malt extract, 5 g/L yeast extract and 50 mL/L glycerol) were maintained in Erlenmeyer flasks under 150 rpm agitation at 28°C.

### Method details

#### *M. perniciosa* mycelial growth in the presence of azoxystrobin

To evaluate the effects of long-term exposure of *M. perniciosa* to azoxystrobin, the fungicide Amistar WG (Syngenta; 50% of azoxystrobin and 50% inert compounds) was added to solid MYEA medium at a gradient of azoxystrobin concentrations: 1 mg/L, 10 mg/L, 20 mg/L, 50 mg/L, 100 mg/L, 200 mg/L and 500 mg/L. MYEA medium with no fungicide served as the control. Fungal growth was evaluated over a 28-day period, with photos and diameter measurements of each culture taken at regular intervals (7-, 14-, 21- and 28-day post inoculation). To evaluate the effects of other strobilurins, metominostrobin and picoxystrobin were tested separately on MYEA medium, using concentration gradients of 1 mg/L, 10 mg/L, 25 mg/L, 50 mg/L, 100 mg/L, 150 mg/L, and 200 mg/L. Each experiment included a fungicide-free MYEA control and followed the same 28-day evaluation protocol.

#### Evaluation of the *M. perniciosa* transcriptome in response to azoxystrobin

We evaluated the early effects of azoxystrobin on the *M. perniciosa* transcriptome using a time-course experiment spanning the first 8 h of exposure to the fungicide. Three biological replicates of each isolate (FA553, FDS01 and FDS02) were cultivated separately in MYEA medium for 28 days. Then, fungal mycelia were transferred to liquid culture and incubated at 28°C with agitation at 150 rpm. After seven days, 2-g aliquots from each replicate were transferred to 20 mL of Malt light liquid medium, either without fungicide (control) or supplemented with 50 mg/L azoxystrobin (azoxystrobin treatment). Samples were harvested for RNA extraction at five time points: 0 min (i.e., immediately after fungal inoculation into the culture medium), 30 minutes, 2 hours, 4 hours, and 8 hours. Figure S1 provides an overview of the experimental design. All samples were processed simultaneously and maintained under identical growth conditions.

#### RNA extraction and RNA-seq library preparation and sequencing

RNA isolation was performed using the RNeasy Plant Kit (QIAGEN, Germany) following the manufacturer’s instructions. RNA integrity and concentration were evaluated using the Lab Chip RNA high sensitivity assay (Caliper, USA). A total of 90 RNA-seq libraries (30 for each isolate) were prepared from 1 μg of total RNA per sample, according to the TruSeq mRNA Stranded Sample Prep Kit protocol (Illumina, USA). The Sciclone G3 Automated Liquid Handling Workstation (PerkinElmer) was used to prepare all libraries simultaneously. Quality control and final library concentrations were assessed using the DNA 1K Lab Chip high sensitivity assay (Caliper, USA). Each individually barcoded library was combined into a single pool and sequenced across nine lanes of an Illumina HiSeq2500 instrument, generating an average of 13.9 million 50 bp single-end sequences per library. The RNA-seq data generated in this study is available at the NCBI Gene Expression Omnibus (GEO) under the accession number GEO: GSE281565.

#### Read mapping

Quality of the raw reads was initially assessed using FASTX-Toolkit v0.0.13.2.^94^ Reads containing Illumina adapter sequences were identified and removed using Cutadapt v1.3.^95^ High-quality reads were then aligned to the *M. perniciosa* genome, obtained from the Witches’ Broom Genome Project (www.lge.ibi.unicamp.br/vassoura), using HiSAT2 v2.1.0.^96^ A maximum of one mismatch was allowed and reads mapping to multiple positions in the reference with equal alignment score were discarded. HTSeq v0.6.1p1^97^ was used to count reads aligning to each of the 17,008 gene models.

#### Hierarchical clustering and principal component analyses

Principal Component Analysis (PCA) was conducted with the AMOR package v0.2-2 in R (https://github.com/surh/AMOR), using the log_2_-transformed expression values (TPM, transcript per million) of the top 500 genes with the highest variance among samples. Hierarchical clustering analyses were performed with the ComplexHeatmap package v2.16.0 in R.^98^ Gene expression values (TPM) were normalized by z-score transformation and clustered based on the Euclidean distance and the complete-linkage method.

#### Functional classification of *M. perniciosa* genes

Functional annotations based on Gene Ontology (GO), InterPro (IPR) terms and TCDB classification (transporter classification database) were previously defined for each of the 17,008 *M. perniciosa* predicted genes.^92^ The annotation of transporters, glutathione transferases and cytochromes P450 were manually curated in this work. Enrichment analyses of GO and InterPro terms were conducted using the BinGO tool v3.0.5 of Cytoscape v3.10.1.^99^^,^^100^ A significance threshold of FDR < 0.05 was used in these enrichment analyses.

#### Identification of genomic variants in *M. perniciosa* genomes

To identify mutations potentially associated with the robust resistance to azoxystrobin in the FDS01 isolate, we employed a comprehensive pipeline for genomic variant calling in *M. perniciosa*. Whole-genome shotgun sequencing was performed on the FA553, FDS01, and FDS02 isolates using mycelial samples from the same cultures as those used in the RNA-seq experiment (Figure S1). Additionally, an extra sample from the original FDS01 culture, along with its parental strain (FA553), were sequenced as temporally spaced replicates, providing further validation of the genomic data.

Genomic DNA was extracted following the protocol described by Mondego et al., (2008). DNA concentration was assessed using the Qubit DNA BR assay kit before library preparation. Libraries were constructed using 200 ng of genomic DNA with the TruSeq DNA Nano Kit (Illumina, USA). Paired-end sequencing (2 × 100 bp) was conducted on the Illumina HiSeq 2500 platform at an average depth of 6.5 million paired-reads per sample, targeting approximately 30× coverage of the *M. perniciosa* genome. Raw sequencing reads were subjected to quality control using FastQC v0.11.8 (Babraham Bioinformatics, UK). Adapter sequences and low-quality bases were trimmed using Trimmomatic v0.36,^101^ retaining only reads with a minimum length of 60 bp for further analysis. Clean reads were aligned to the *M. perniciosa* FA553 reference genome using Bowtie2 v2.4.0^102^ with default parameters. Genomic variants, including single nucleotide polymorphisms (SNPs) and small insertions/deletions (INDELs), were identified using two independent tools: FreeBayes v1.1.0^103^ and BCFtools mpileup v1.11.^104^ After marking duplicate reads using Picard Tools v2.23.4 (Broad Institute, USA), variant calling was performed for all five genomes. BCFtools isec v1.11 was used to identify variants unique to the FDS01 genome. Finally, variants called by FreeBayes were filtered using VcfFilter v0.2 with the following criteria: “QUAL > 20 & QUAL / AO > 10 & SAF > 1 & SAR > 1 & RPR > 1 & RPL > 1.” Variants identified by mpileup were filtered using SnpSift filter v4.3t^105^ with the criteria: “QUAL >= 20 && DP > 5 && MQ > 35.” The resulting variants were manually curated and categorized by their genomic location (exonic, intronic, intergenic) and their potential impact (e.g., synonymous, nonsynonymous, frameshift).

### Quantification and statistical analysis

Differential expression analysis was performed with the edgeR package v3.18.1,^19^ applying the False Discovery Rate (FDR) method^106^ for multiple-testing correction. To filter out weakly expressed genes, only those with a minimum expression level of 1 count per million in at least three libraries were included in the analysis. Normalization was performed using the trimmed mean of M-values method (TMM; function calcNormFactors in edgeR).^107^ Count data were then fit into a negative binomial generalized linear model with a log link function. The experimental design followed a one-way layout and included ten groups representing the combinations of the two treatments in five time points. Contrasts were made between treated samples and controls at each time point and genes with FDR<0.01 and a fold-change of at least 1.5× were considered differentially expressed.

## Supplemental information

Document S1. Figures S1–S6

Table S1. Differential expression analysis of *M. perniciosa* genes

Table S2. Gene Ontology (GO) enrichment analysis following azoxystrobin treatment

Table S3. Fold change and FDR values for genes involved in metabolic processes

Table S4. Fold change and FDR values for genes involved in basic biological processes

Table S5. Fold change and FDR values for genes involved in detoxification mechanisms

Table S6. Comparisons of gene expression profiles between *M. perniciosa* isolates

Table S7. Gene Ontology (GO) enrichment analysis based on the contrasts between the transcriptional profiles of *M. perniciosa* isolates

Table S8. Identified genetic variants in FDS01 *M. perniciosa* isolate compared to the FA553 isolate
