# Supplementary material for: Unveiling molecular mechanisms of strobilurin resistance in the cacao pathogen Moniliophthora perniciosa
Source: iScience. 2025 Jul 23;28(8):113180. doi: 10.1016/j.isci.2025.113180 (PMC12432460; doi:10.1016/j.isci.2025.113180)
Supplement: Document S1. Figures S1–S6 [file mmc1.pdf]

iScience, Volume *28*

Supplemental information

Unveiling molecular mechanisms of strobilurin resistance in the cacao pathogen *Moniliophthora perniciosa*

Paula F.V. Prado, Caio V.C. Mendes, Bárbara A. Pires, Gabriel L. Fiorin, Piotr Mieczkowski, Gonçalo A.G. Pereira, Paulo J.P.L. Teixeira, and Daniela P.T. Thomazella

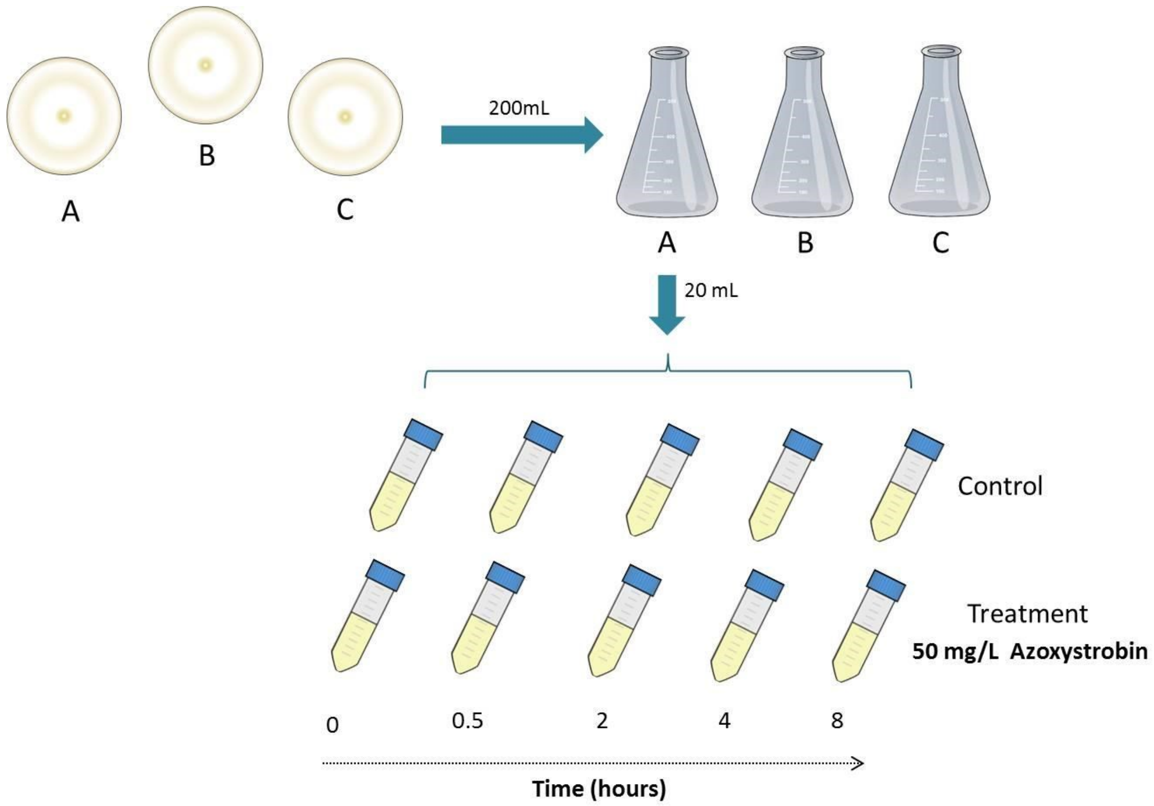

Figure S1. Experimental design for *M. perniciosa* treatment with azoxystrobin in a time-course experiment, related to STAR Methods. Three biological replicates were inoculated in liquid Malt light medium. Samples for RNA sequencing were collected at five time points after azoxystrobin treatment, along with corresponding control samples (without fungicide). The experiment comprises a total of 30 individual RNA-seq libraries per isolate.

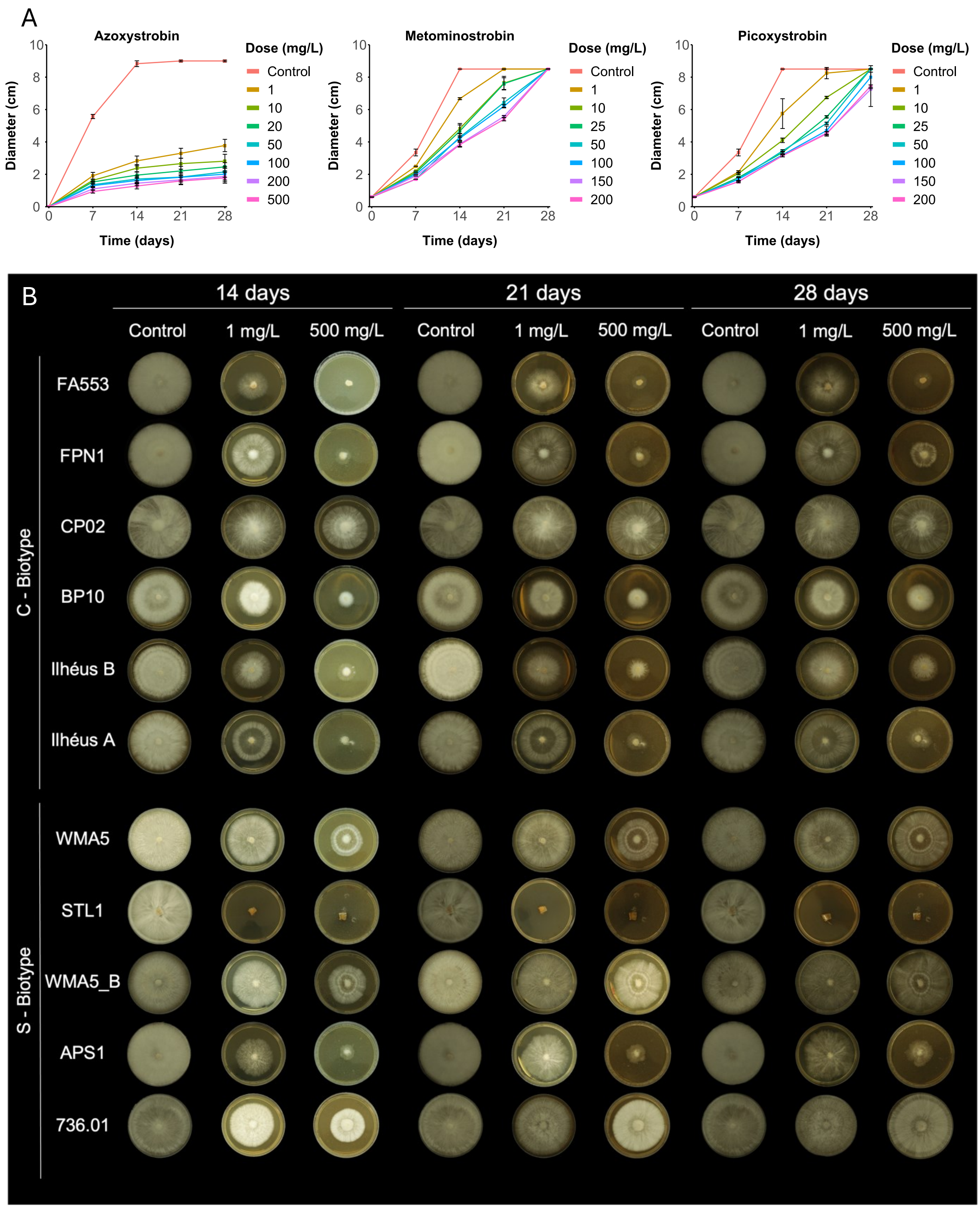

Figure S2. Evaluation of *M. perniciosa* growth in the presence of strobilurins, related to Figure 1. A. Growth of *M. perniciosa* in the presence of the strobilurins azoxystrobin, metominostrobin, and picoxystrobin. The diameter of fungal colonies was measured over 28 days at different concentrations of each fungicide: azoxystrobin (left), metominostrobin (center) and picoxytrobin (right). Data are represented as mean and error bars indicate the standard error (n= 3). Under control conditions (i.e., without fungicide), the mycelium covered the entire plate (9 cm in diameter) within approximately 14 days, leading to the plateau observed in the graphs. B. Growth of different *M. perniciosa* isolates in the presence of azoxystrobin. Isolates were cultivated for 28 days on medium supplemented with 0 mg/L (control), 1 mg/L or 500 mg/L azoxystrobin. All isolates exhibited fungal growth at both concentrations, except STL1 (S-biotype), which is susceptible to the fungicide. Although growth was reduced in a dose-dependent manner, even 500 mg/L azoxystrobin did not fully inhibit *M. perniciosa* development. Representative images from three replicates are shown.

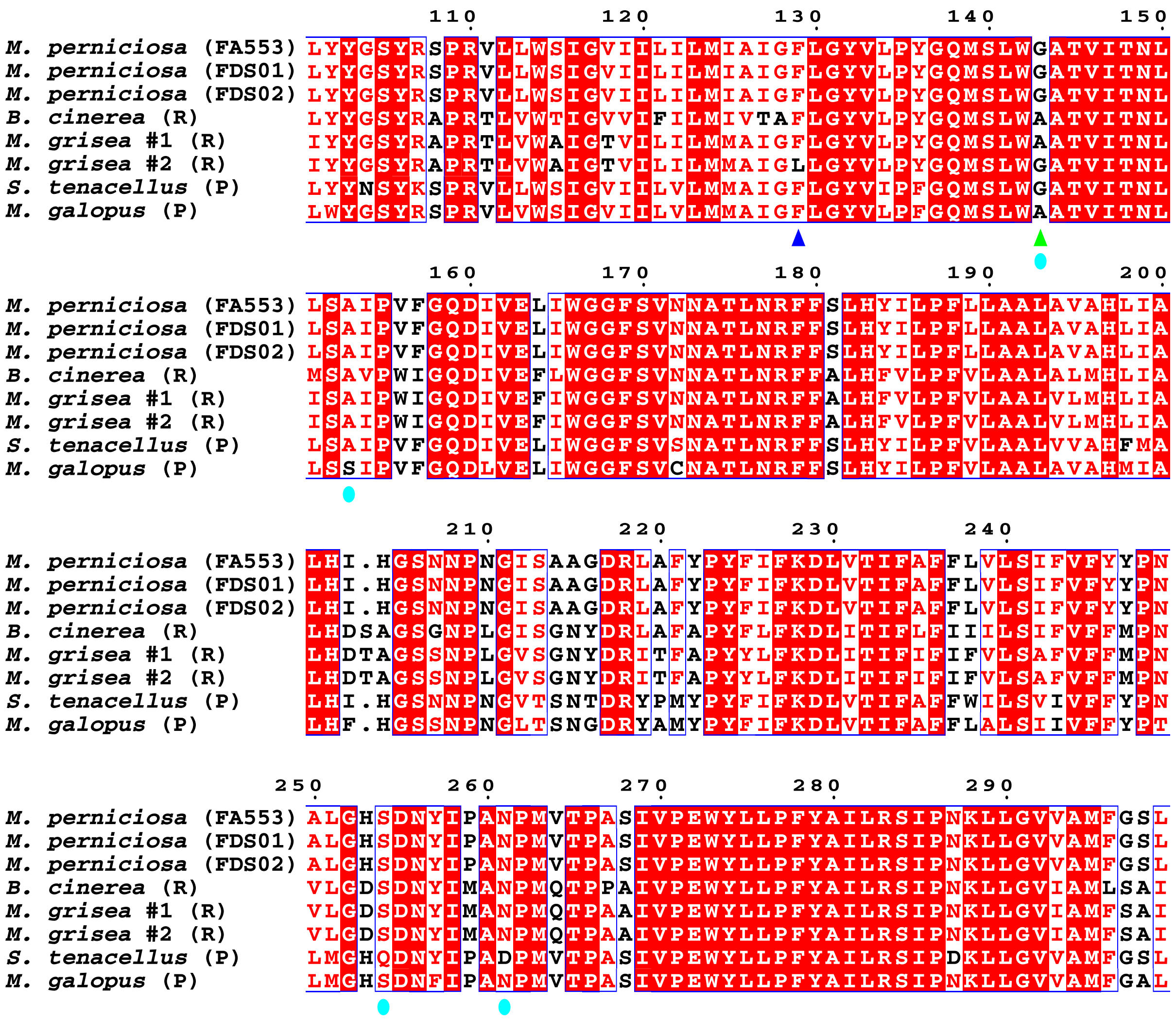

Figure S3. Mutations in the *CytB* gene that determine azoxystrobin resistance in phytopathogens are absent in *M. perniciosa*, related to Figures 2, 7 and 8. Alignment of a 300-amino acid fragment of the CytB (cytochrome B) protein from different fungi. Dark blue and green arrows correspond to the F129L and G143A mutations, respectively, which are known to confer strobilurin resistance in pythopathogens (R). Cyan circles indicate mutations in the CytB protein that have been identified in strobilurin-producing basidiomycetes (P) and confer natural resistance to their own product. None of the *M. perniciosa* isolates used in this study contain any of these alterations. Protein sequences were retrieved from the NCBI database with the following IDs: *Botrytis cinerea* (ACL50592.1); *Magnaporthe grisea* G143A mutant (AAO91631.1); *Magnaporthe grisea* F129L mutant (AAO91630.1); *Strobilurus tenacellus* (CAA61250.1); *Mycena galopus* (CAA61247.1).

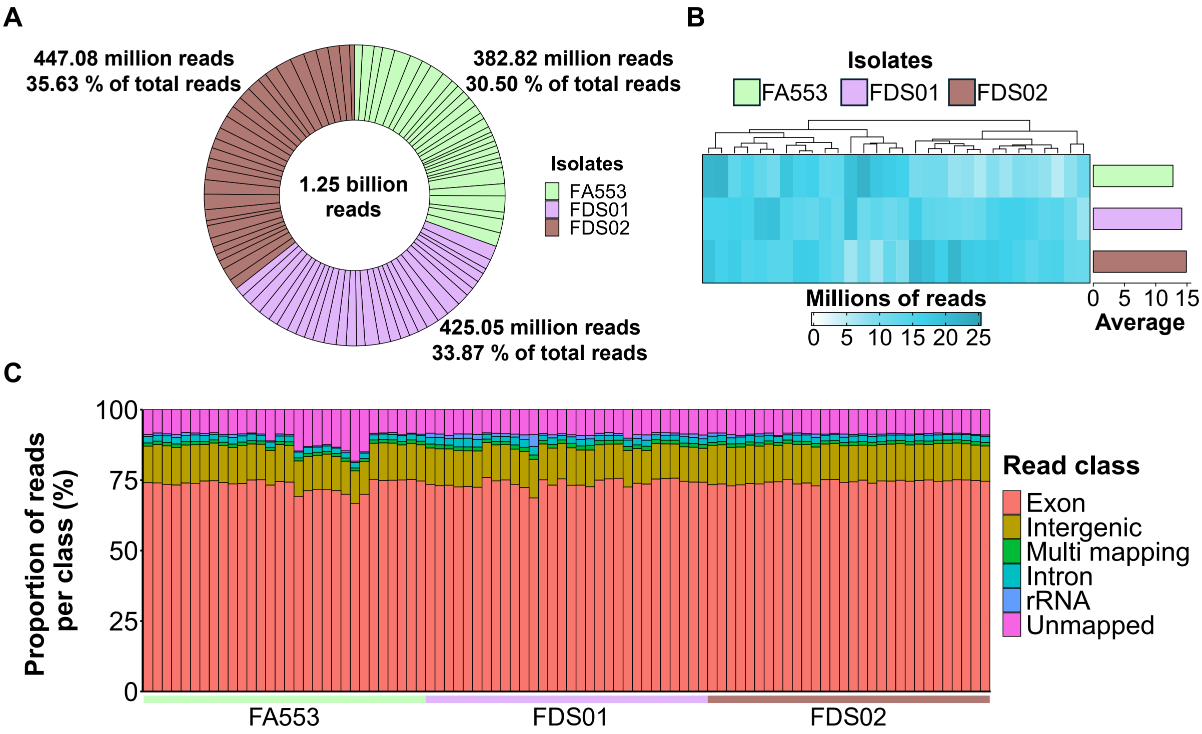

Figure S4. Sequencing and mapping metrics, related to Figures 2 and 7. (A) Distribution of the 1.25 billion reads generated for the 90 RNA-seq libraries from three *M. perniciosa* isolates (FA553, FDS01 and FDS02). Each sector in the chart represents an individual library. (B) Heatmap showing the number of reads per RNA-seq library. Each row corresponds to one *M. perniciosa* isolate, and the bar plots on the right show the average number of reads produced for each library. (C) Percentage of reads mapped to intergenic and genic regions (exon and intron), multi-mapped to more than one genomic position, mapped to rRNA and unmapped reads (total reads passing QC minus mapped reads). Each library is represented by a column.

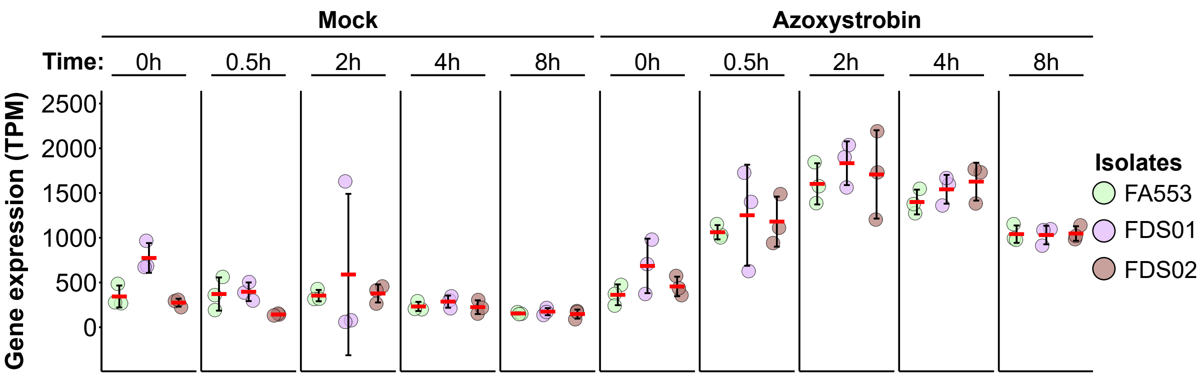

Figure S5. Up-regulation of alternative oxidase (*Mp-Aox*) is not linked to the increased tolerance of the FDS01 isolate to azoxystrobin, related to Figures 7 and 8. *Mp-Aox* is similarly up-regulated in all three *M. perniciosa* isolates at all time points. Error bars indicate the standard error (n= 3).

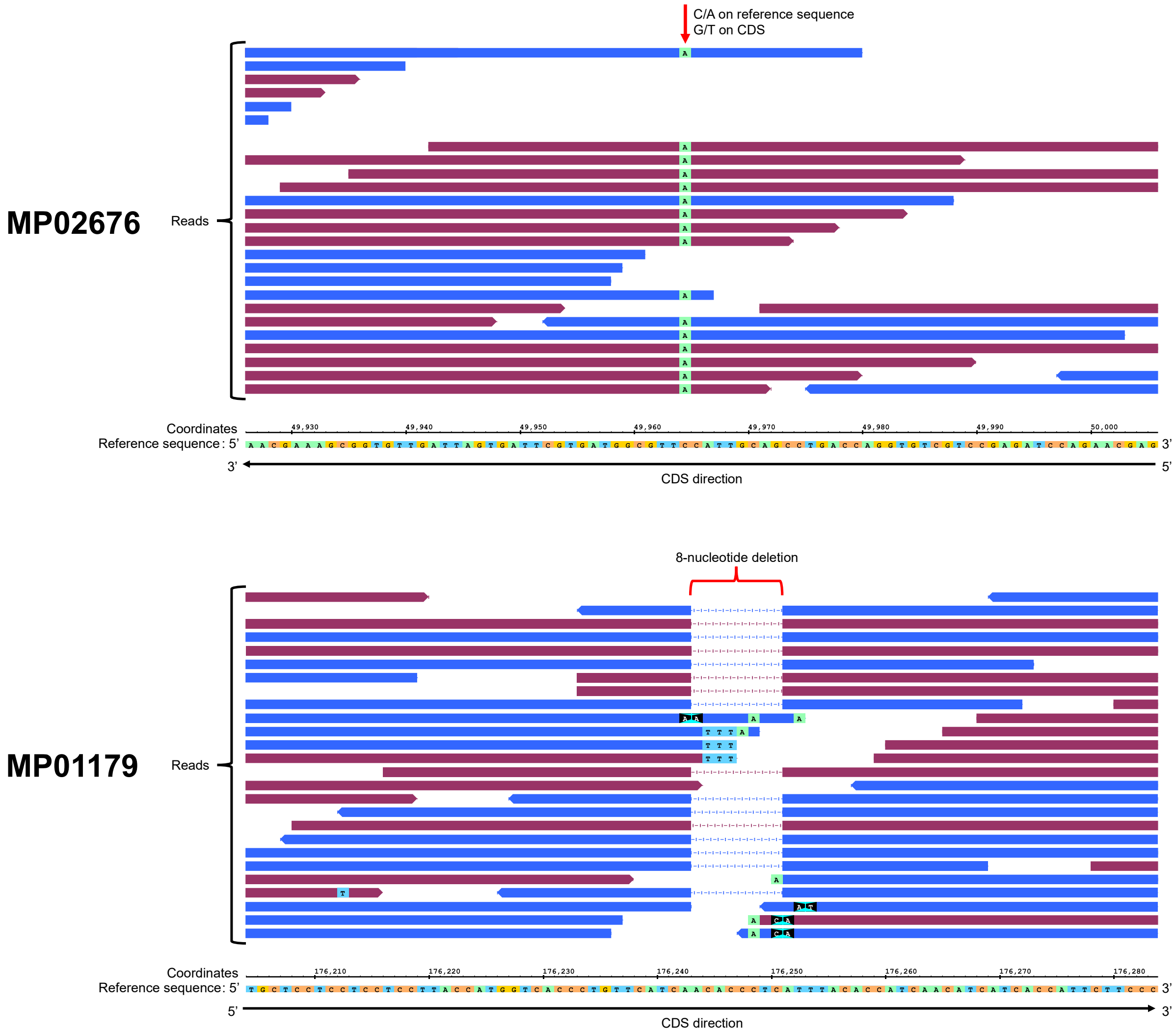

Figure S6. Genome sequencing reveals mutations in two genes of the FDS01 isolate, related to Figure 8. Sequenced reads of the MP02676 gene from the FDS01 genome show the occurrence of a SNP (C to A in the reference sequence and G to T in the reference CDS) compared to the FA553 reference genome. Additionally, gaps in the FDS01 genomic reads indicate an 8-nucleotide deletion in the MP01179 gene.
